# *KCNQ1OT1* promotes ovarian cancer progression via modulating *MIR‐142‐5p*/*CAPN10* axis

**DOI:** 10.1002/mgg3.1077

**Published:** 2020-01-07

**Authors:** Hongli Liu, Ruixin Chen, Fenhong Kang, Haiqing Lai, Yanlong Wang

**Affiliations:** ^1^ Department of Gynecology, Women and Children’s Hospital School of Medicine Xiamen University Fujian China

**Keywords:** *CAPN10*, *KCNQ1OT1*, *MIR‐142‐5p*, ovarian cancer

## Abstract

**Background:**

Long non‐coding RNA (lncRNA) has been regarded as crucial regulator for cancer progression. Roles of KCNQ1 opposite strand/antisense transcript 1 (*KCNQ1OT1*) in cancers including osteosarcoma and colon cancer have been previously reported. However, its role in ovarian cancer (OC) remains unclear.

**Methods:**

Expression level of *KCNQ1OT1* on OC cells and normal cell was analyzed with quantitative real‐time PCR. Gain and loss‐of‐function experiments were performed to analyze the biological roles of *KCNQ1OT1* in OC. Moreover, whether *KCNQ1OT1* functions its role via mediating *MICRORNA‐142‐5p* (*MIR‐142‐5p*)/calpain 10 (*CAPN10*) axis was analyzed. In addition, effects of *KCNQ1OT1*, *MIR‐142‐5p*, and *CAPN10* on overall survival of OC patients were analyzed at Kaplan–Meier plotter website.

**Results:**

We showed *KCNQ1OT1* was elevated expression in OC cells and indicated poorer overall survival of OC patients. Besides, we found *KCNQ1OT1* could promote OC cell proliferation and migration in vitro. Moreover, *MIR‐142‐5p* was found reduced expression, while *CAPN10* was found elevated expression in OC cells compared with normal cell. Kaplan–Meier curve analysis showed low *MIR‐142‐5p* or high *CAPN10* expression were indicators for poorer overall survival of OC patients. At length, we showed *KCNQ1OT1* could regulate OC development via *MIR‐142‐5p*/*CAPN10* axis.

**Conclusions:**

Taken together, *KCNQ1OT1* upregulates *CAPN10* expression via sponging *MIR‐142‐5p*, thus promoting the proliferation and migration of OC.

## Graphical abstract

KCNQ1OT1 expression was revealed highly expressed in GC cells. High KCNQ1OT1 expression was a predictor for poor overall survival of cancer patients.

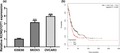

## 1 INTRODUCTION

Ovarian cancer (OC) is a common occurred cancer type in female reproductive system (Siegel, Miller, & Jemal, 2018). The 5‐year overall survival for OC patients is about 30% due to approximately 75% of cancer patients are diagnosed at late stages (Siegel, Miller, & Jemal, 2017; Sorensen, Schnack, Karlsen, & Hogdall, 2015). Therefore, it is imperative to explore the novel targets which can be used for tumor‐targeted therapy.

Emerging evidence showed OC carcinogenesis is correlated with aberrantly expressed of long non‐coding RNAs (lncRNAs) (Wang, Lu, & Chen, 2019). Even though lncRNA lacks the ability to code proteins, it was reported to regulate various cellular processes and tumorigenesis (Bartonicek, Maag, & Dinger, 2016). For instance, lncRNA FLVCR1 antisense RNA 1 (*FLVCR1‐AS1*) was reported to overexpressed in OC tissues, serum, and cells (Yan et al., 2019). The overexpression of *FLVCR1‐AS1* was revealed to promote cancer proliferation, metastasis, and epithelial to mesenchymal transition via regulating *Yes associated protein 1* (606608) expression via modulating *MICRORNA‐513*, while the knockdown of *FLVCR1‐AS1* caused opposite effects on OC cell behaviors (Yan et al., 2019). Moreover, lncRNA growth arrest specific 5 (*GAS5*, 608280) was identified to be elevated expression in epithelial ovarian cancer tissues and cells (Long, Xiong, et al., 2019). Mechanism study showed that *GAS5* could recruit transcription factor enhancer of zeste 2 polycomb repressive complex 2 subunit (601573)to regulate map kinase signal pathway (Long, Song, et al., 2019).

Potassium Voltage‐Gated Channel Subfamily Q Member 1 opposite strand/antisense transcript 1 (*KCNQ1OT1,* 604115) is a long non‐coding RNA located at chromosome 11p15.5 (Mitsuya et al., 1999). *KCNQ1OT1* was revealed to be elevated expression in colon and rectal adenocarcinoma (Zhang, Yan, Yi, Rui, & Hu, 2019). Also, high *KCNQ1OT1* was found to be a predictor for poorer overall survival and recurrence‐free survival of colon adenocarcinoma patients (Zhang et al., 2019). Moreover, *KCNQ1OT1* was found could regulate the response of cancer cells to chemo‐reagents. For example, *KCNQ1OT1* was found overexpressed in methotrexate resistant colorectal cancer cells (Xian & Zhao, 2019). Also, they showed *KCNQ1OT1* was able to affect the chemosensitivity of colorectal cancer cell via targeting protein phosphatase 1 regulatory inhibitor subunit 1B (604399) through sponging *MIR‐760* (Xian & Zhao, 2019). In colon cancer, *KCNQ1OT1* was also identified to be elevated expression and correlated with poor overall survival of cancer patients (Li et al., 2019). Moreover, it was found knockdown of *KCNQ1OT1* inhibits cell proliferation but promotes cell apoptosis through *MIR‐34a* (611172)/autophagy‐related 4B cysteine peptidase (611338) (Li et al., 2019). Besides that, KCNQ1OT1 was shown to regulate the osteosarcoma cell behaviors and its response to cisplatin via regulating Kcnq1/DNA methyltransferase 1 mediated KCNQ1 expression (Li et al., 2019). However, its expression and roles of *KCNQ1OT1* in OC remains to be explored.

In this study, expression of *KCNQ1OT1* in OC tissues and cell lines was explored. Moreover, a series of loss and gain‐of‐function experiments were performed to investigate the biological roles of *KCNQ1OT1*. Moreover, the underlying mechanism of *KCNQ1OT1* in regulating OC cell behaviors was explored.

## 2 MATERIALS AND METHODS

### 2.1 Cell culture

The Roswell Park Memorial Institute‐1640 (RPMI‐1640) and fetal bovine serum (FBS) purchased from Invitrogen (Thermo Fisher Scientific, Inc., Waltham, MA, USA) was used to incubate OC cells (SKOV3, OVCAR3) and normal ovarian epithelial cell line (IOSE80). The incubation condition was maintained at 37°C and contained 5% of CO_2_. All these three cells were obtained from American Type Culture Collection (ATCC).

### 2.2 Cell transfection

For manipulating the expression of *KCNQ1OT1*, small‐interfering RNA (siRNA) for *KCNQ1OT1* (si‐*KCNQ1OT1*, 5ʹ‐GGTAGAATAGTTCTGTCTT‐3ʹ), overexpression construct for KCNQ1OT1 (pKCNQ1OT1) or calpain 10 (605286, pCAPN10), and the relative controls (siR‐NC, 5ʹ‐ATCTATGATTGGTCTATGG‐3ʹ and pcDNA3.1) was synthesized by GenScript. MIR‐140‐5p mimic (5ʹ‐CAGUGGUUUUACCCUAUGGUAG‐3ʹ) and the corresponding negative control (miR‐NC, 5ʹ‐GGUCAUCGGAUUGUAUCUCGUA‐3ʹ) were designed by GenePharm. Cells were transfected using Lipofectamine 2000 (Invitrogen) until incubated to approximately 80% confluence according to the standard protocols.

### 2.3 Quantitative real‐time PCR (qRT‐PCR)

To explore relative gene expression level, qRT‐PCR was performed on ABI 7,500 system (Applied Biosystem). RNA from cells was extracted using Trizol reagent (Invitrogen) and then reverse transcribed into complementary DNA using First‐Strand cDNA Synthesis kit (Invitrogen). qRT‐PCR was performed using SYBR Green Mix (Takara, Dalian, Liaoning, China) with the following procedure: 95°C for 10 min, 40 cycles of 95°C for 10 s, 55°C for 10 s, and 72°C for 30 s. Relative expression level was calculated with the 2−ΔΔCt method. Primers used were as follows: KCNQ1OT1: forward: 5ʹ‐CCTCCCTCACTGAGCTTTGG‐3ʹ, reverse: 5ʹ‐GTGCGGACCCTATACGGAAG‐3ʹ; *CAPN10*: forward: 5ʹ‐GCTGGCTGGTGACATCAGTG‐3ʹ, reverse: 5ʹ‐TCAGGTTCCATCTTTGGGCCAG‐3ʹ; glyceraldehyde‐3‐phosphate dehydrogenase (138400): forward: 5ʹ‐GCTGCTGAGTATGTCGTGGAGT‐3ʹ, reverse: 5ʹ‐AGTCTTCTGGGTGGCAGTGAT‐3ʹ; *MIR‐142‐5p*: forward: 5ʹ‐CAUAAAGUAGAAAGCACUACU‐3ʹ, reverse: 5ʹ‐UAGUGCUUUCUACUUUAUGUU‐3ʹ; *U6 snRNA*: forward: 5ʹ‐CTCGCTTCGGCAGAC‐3ʹ, reverse: 5ʹ‐AACGCTTACGAATTT‐3ʹ. The procedures were as follows: 95°C for 3 min, 40 cycles of 95°C for 10 s, and 58°C for 30 s.

### 2.4 Cell counting kit‐8 (CCK‐8) assay

Cell proliferation ability was analyzed with CCK‐8 (Beyotime, Haimen, Jiangsu, China) assay according to the supplier's instructions. Cell proliferation rate was analyzed at 0, 1, 2, and 3 days after incubation. At these time points, CCK‐8 reagent was added to each well and then the optical density at 450 nm was measured using microplate reader.

### 2.5 Colony formation assay

Eight hundred cells were seeded into 6‐well plate and incubated for 14 days. Then, methanol was added to the plate to fix the colonies. Colonies were further stained with crystal violet and counted under microscope.

### 2.6 Transwell invasion assay

Cell invasion ability was analyzed with transwell invasion assay. 2 × 10^5^ in serum‐free medium was added into the upper chamber of 8 μm transwell unit (Corning, NY, USA) that pre‐coated with Matrigel. RPMI‐1640 supplemented with FBS was filled into the lower chamber. After incubation for 48 hr, invasive cells were fixed and stained. At length, the invasive cell numbers were counted under microscope.

### 2.7 Luciferase reporter assay

lncBase was used to explore the potential miRNA target of *KCNQ1OT1*, and we identified *MIR‐142‐5p* was a potential target. TargetScan was utilized to investigate the potential target of *MIR‐142‐5p*, and we showed *CAPN10* was a putative target. The wild‐type sequence of *KCNQ1OT1* and *CAPN10* was inserted into pmirGLO to generate *KCNQ1OT1*‐wt and *CAPN10*‐wt. Site‐direct mutagenesis kit was used to generate mutant luciferase vectors (*KCNQ1OT1*‐wt and *CAPN10*‐wt). Cells were co‐transfected with luciferase vectors or miRNAs using Lipofectamine 2000. After 48 hr of transfection, relative luciferase activity was analyzed with Dual‐luciferase reporter assay system (Promega, Madison, WI, USA).

### 2.8 Prognostic values of *KCNQ1OT1*, *MIR‐142‐5p*, and *CAPN10* on OC patients

The effects of *KCNQ1OT1*, *MIR‐142‐5p*, and *CAPN10* on OC patients were explored at Kaplan‐Meier plotter website (http://kmplot.com/analysis/index.php?p=background, Nagy, Lánczky, Menyhárt, & Győrffy, 2018).

### 2.9 Statistical analysis

Results obtained from in vitro experiments were analyzed at Graphpad prism 7 (San Diego, CA, USA) and then presented as mean ± standard deviation (*SD*). Differences among groups were analyzed using Student's *t*‐test or one‐way Analysis of Variance and Tukey post‐hoc test. *p* < .05 was believed as statistically significant.

## 3 RESULTS

### 3.1 Expression of *KCNQ1OT1* was elevated in OC

**Figure 1:**
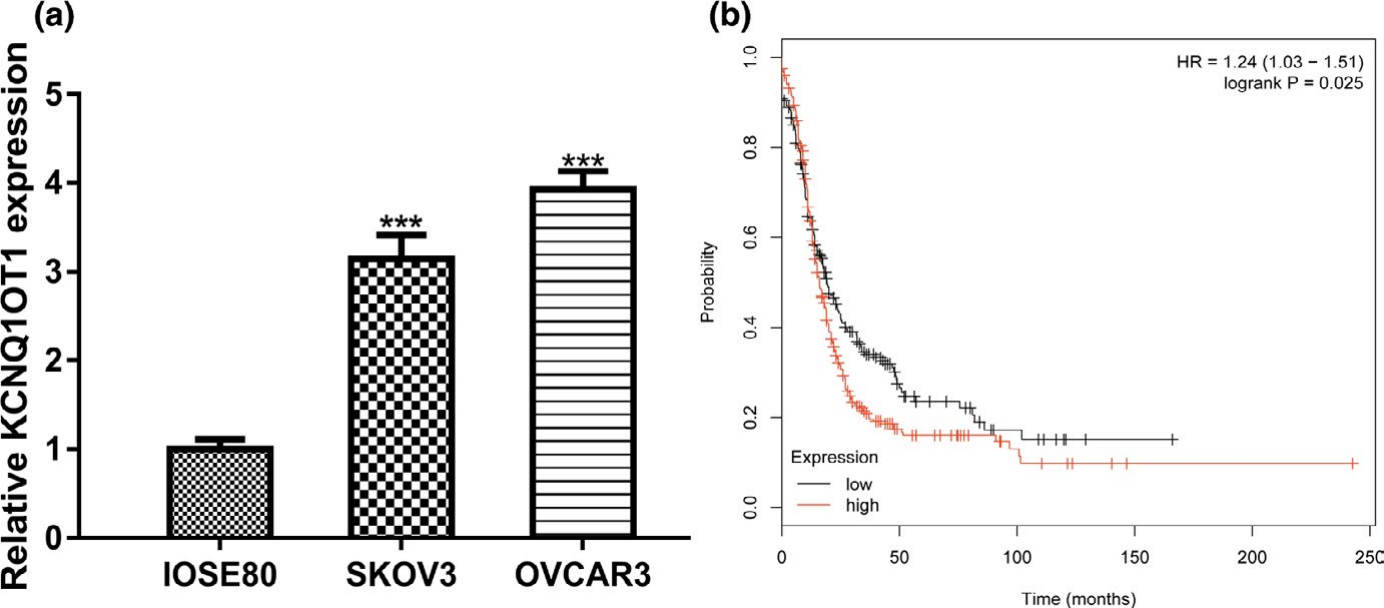
*KCNQ1OT1* expression and its impact on overall survival of OC patients. (a) Expression of *KCNQ1OT1* in OC cells and normal cell line. (b) Effect of *KCNQ1OT1* on the overall survival of OC patients. *KCNQ1OT1*, Potassium Voltage‐Gated Channel Subfamily Q Member 1 opposite strand/antisense transcript 1; OC, ovarian cancer

qRT‐PCR revealed that *KCNQ1OT1* levels were significant higher in OC cell lines than in normal cell line (Figure 1a). Survival analysis revealed that patients with high *KCNQ1OT1* expression level tend to have worse prognosis compared to those with low *KCNQ1OT1* expression level (Figure 1b).

### 3.2 *KCNQ1OT1* overexpression promotes OC cell proliferation and invasion in vitro

**Figure 2:**
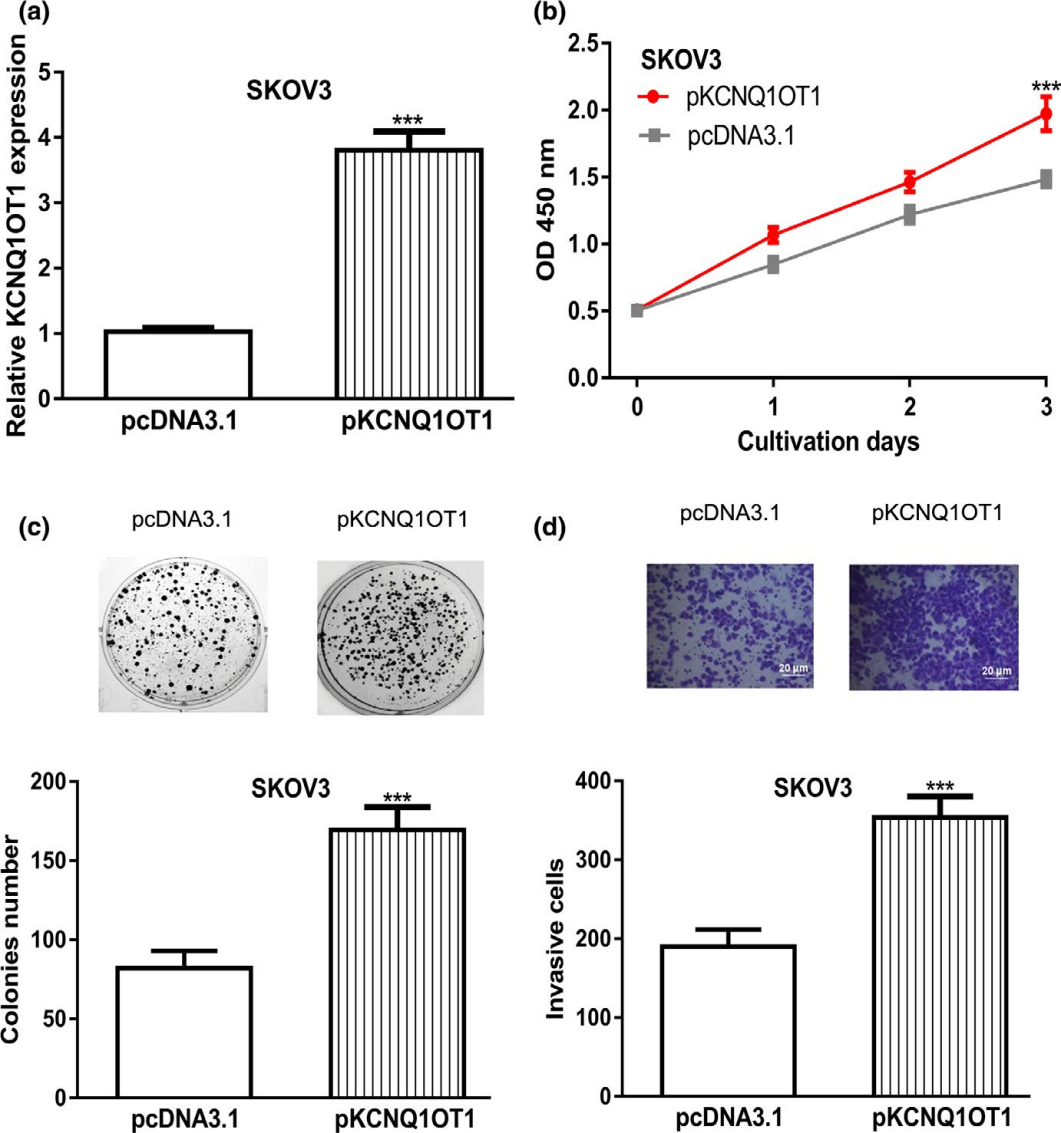
*KCNQ1OT1* overexpression promoted cell proliferation, colony formation, and invasion. (a) *KCNQ1OT1* expression was detected following pKCNQ1OT1 transfection. (b) CCK‐8 assay to detect proliferation of cells transfected with pKCNQ1OT1. (c) Colony formation of cells after transferring of pKCNQ1OT1. (d) Cell invasion rate of cells with pKCNQ1OT1 transfection was measured. CCK‐8: cell counting kit‐8; *KCNQ1OT1*, Potassium Voltage‐Gated Channel Subfamily Q Member 1 opposite strand/antisense transcript 1; OC, ovarian cancer

The SKOV3 cell line was selected for gain‐of‐function experiments. qRT‐PCR revealed that *KCNQ1OT1* levels could be significantly elevated by pKCNQ1OT1 (Figure 2a). CCK‐8 assay and colony formation assay revealed that *KCNQ1OT1* overexpression promoted OC cell growth (Figure 2b,c). Furthermore, transwell invasion assay showed the cell invasion of OC cell was promoted after *KCNQ1OT1* overexpression (Figure 2d).

### 3.3 Knockdown of *KCNQ1OT1* inhibits OC cell proliferation and invasion in vitro

**Figure 3:**
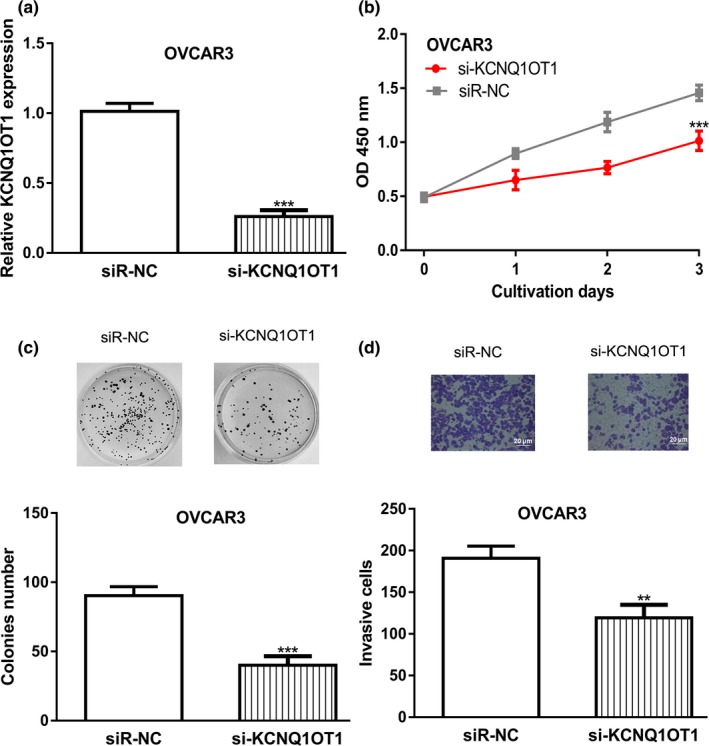
*KCNQ1OT1* knockdown inhibited cell proliferation, colony formation, and invasion. (a) *KCNQ1OT1* expression was detected following si‐KCNQ1OT1 transfection. (b) CCK‐8 assay to detect proliferation of cells transfected with si‐KCNQ1OT1. (c) Colony formation of cells after transferring of si‐KCNQ1OT1. (d) Cell invasion rate of cells with si‐KCNQ1OT1 transfection was measured. CCK‐8: cell counting kit‐8; *KCNQ1OT1*, Potassium Voltage‐Gated Channel Subfamily Q Member 1 opposite strand/antisense transcript 1; OC, ovarian cancer; siR‐NC, negative control small interfering RNA

OVCAR3 cell line was used for loss‐of‐function experiments. si‐KCNQ1OT1 introduction significantly decreased the levels of *KCNQ1OT1* (Figure 3a). In vitro experiments revealed that knockdown the expression *KCNQ1OT1* was able to inhibit OC cell proliferation, colony formation, and cell invasion (Figure 3b‐d).

### 3.4 Interaction of *KCNQ1OT1* and *MIR‐142‐5p* in OC

**Figure 4:**
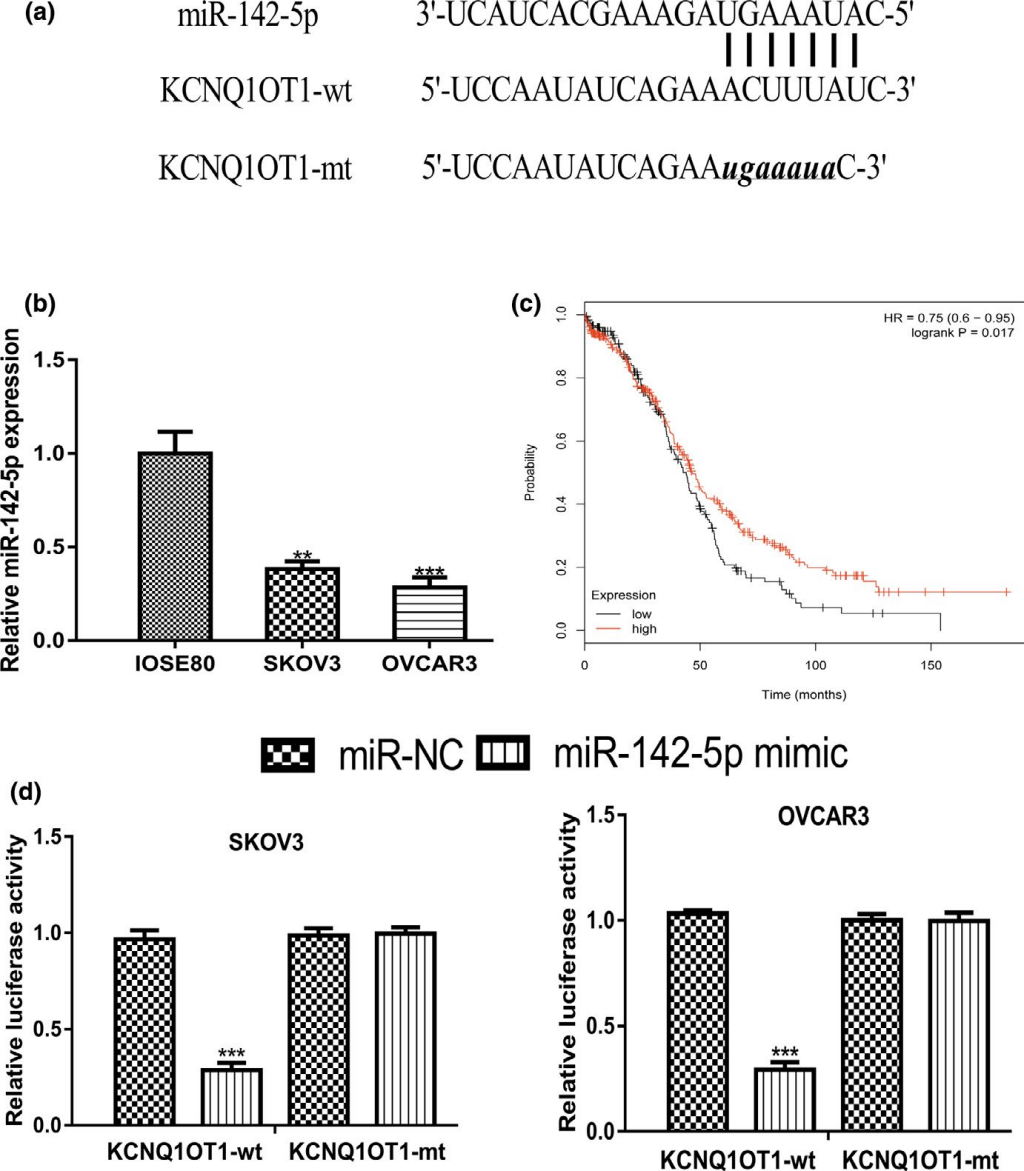
*KCNQ1OT1* functioned as a sponge for *MIR‐142‐5p*. (a) Binding model of *KCNQ1OT1* and *MIR‐142‐5p*. (b) Expression of *MIR‐142‐5p* in OC cells and normal cell line. (c) Effect of *MIR‐142‐5p* on the overall survival of OC patients. (d) Luciferase activity in cells with luciferase vectors and miRNAs transfection. *KCNQ1OT1*, Potassium Voltage‐Gated Channel Subfamily Q Member 1 opposite strand/antisense transcript 1; *MIR‐142‐5p*, MICRORNA‐142‐5p; miR‐NC, negative control microRNA; mt: mutant; OC, ovarian cancer; wt: wild‐type

lncBase showed that *MIR‐142‐5p* was a putative target for *KCNQ1OT1* (Figure 4a). *MIR‐142‐5p* expression level was revealed to be decreased expression in OC cells compared with normal cell line (Figure 4b). Meanwhile, we showed that low *MIR‐142‐5p* was a predictor for poor overall survival of OC patients (Figure 4c). Luciferase activity reporter assay revealed that overexpression of *MIR‐142‐5p* inhibited the luciferase activity of cells transfected with *KCNQ1OT1*‐wt (Figure 4d).

### 3.5 Interaction of *MIR‐142‐5p *and *CAPN10* in OC

**Figure 5:**
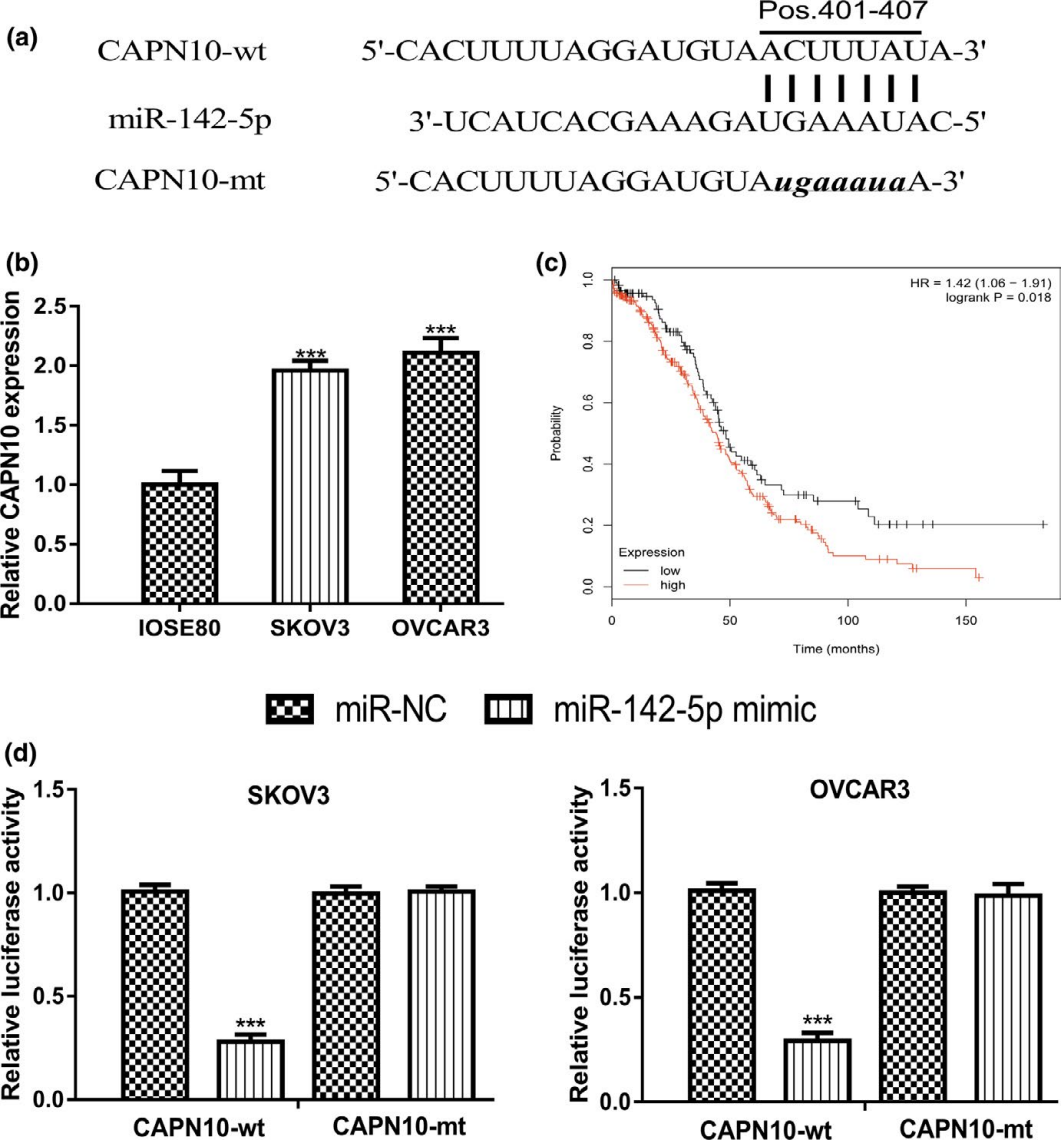
*MIR‐142‐5p* directly target *CAPN10*. (a) Binding model of *CAPN10* and *MIR‐142‐5p*. (b) Expression of *CAPN10* in OC cells and normal cell line. (c) Effect of *CAPN10* on the overall survival of OC patients. (d) Luciferase activity in cells with luciferase vectors and miRNAs transfection. *CAPN10*, calpain 10; *MIR‐142‐5p*, MICRORNA‐142‐5p; miR‐NC, negative control microRNA; mt, mutant; OC, ovarian cancer; wt, wild‐type

Furthermore, TargetScan showed that *CAPN10* was a putative target for *MIR‐142‐5p* (Figure 5a). qRT‐PCR revealed that *CAPN10* expression level in OC cells was higher than in normal cell line (Figure 5b). Kaplan–Meier plotter showed that high *CAPN10* level indicates a poor prognosis of OC cancer patients (Figure 5c). Luciferase activity reporter assay indicated that overexpression of *MIR‐142‐5p* inhibits the relative luciferase activity of cells transfected with *CAPN10*‐wt (Figure 5d).

### 3.6 *KCNQ1OT1* exerts its function through *MIR‐142‐5p*/*CAPN10*

**Figure 6:**
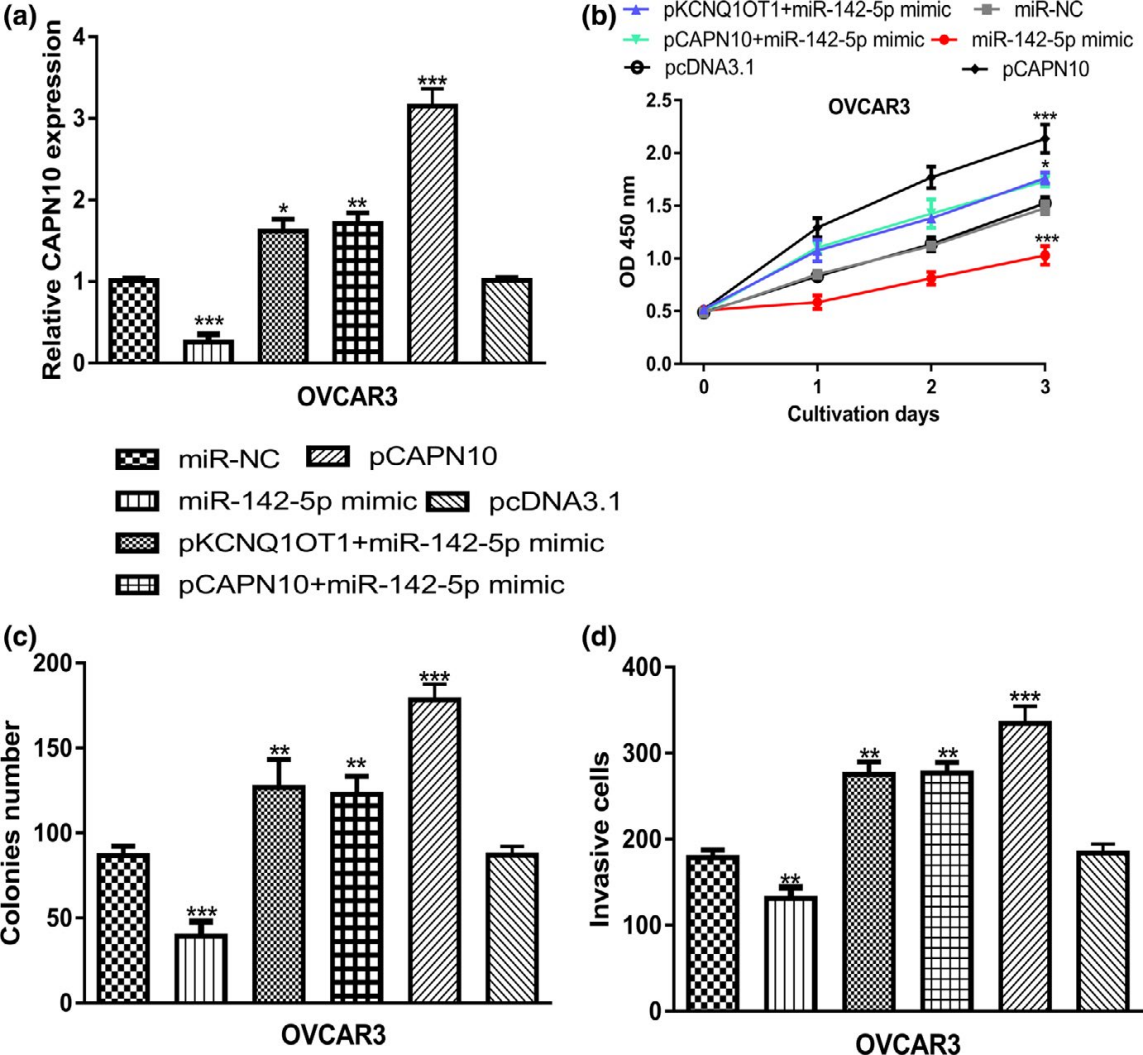
*KCNQ1OT1* regulated OC progression via regulating *MIR‐142‐5p*/*CAPN10* axis. (a) *CAPN10* expression was detected following miRNA or overexpression construct transfection. (b) CCK‐8 assay to detect proliferation of cells transfected with miRNA or overexpression construct. (c) Colony formation of cells after transferring of miRNA or overexpression construct. (d) Cell invasion rate of cells with miRNA or overexpression construct transfection was measured. *CAPN10*, calpain 10; CCK‐8, cell counting kit‐8; *KCNQ1OT1*, Potassium Voltage‐Gated Channel Subfamily Q Member 1 opposite strand/antisense transcript 1; *MIR‐142‐5p*: MICRORNA‐142‐5p; miR‐NC, negative control microRNA; OC, ovarian cancer

qRT‐PCR revealed that *CAPN10* expression was significantly increased by pCAPN10 (Figure 6a). CCK‐8 assay, colony formation, and transwell invasion assay showed that *CAPN10* overexpression promotes OC cell growth and invasion (Figure 6b‐d). In the meantime, we showed overexpression of MIR‐142‐5p inhibits OC cell proliferation, colony formation, and invasion (Figure 6b‐d). Importantly, we showed *KCNQ1OT1* targeting the *MIR‐142‐5p*/*CAPN10* axis to regulate OC cell behaviors through rescue experiments (Figure 6b‐d).

## 4 DISCUSSION

LncRNA and miRNAs are RNA molecules that play crucial roles in multiplies cellular processes including cell growth, metastasis, and drug resistant (Li & Zhan, 2019; Long, Song, et al., 2019; Qi et al., 2019). The competitive RNA (ceRNA) theory has connected lncRNA and miRNA together (Salmena, Poliseno, Tay, Kats, & Pandolfi, 2011). A recent work conducted by Zhao et al. revealed several lncRNA/miRNA/mRNA triplets that may contribute to the cisplatin‐resistant in epithelial OC (Zhao, Tang, Zuo, Zhang, & Wang, 2019). Hence, the investigations on the aberrantly expressed molecules contributed to OC initiation and progression may help to better control OC in the future.

Previous studies have suggested that lncRNA *KCNQ1OT1* plays crucial roles in cancer biology (Li et al., 2019; Xian & Zhao, 2019). In this work, we showed expression levels of lncRNA *KCNQ1OT1* in OC cells were higher than in normal cell line. Through Kaplan–Meier plotter analysis, we showed high *KCNQ1OT1* expression was correlated with poor overall survival of OC patients. Moreover, after *KCNQ1OT1* overexpression, we showed that cell proliferation, colony formation, and cell invasion of OC cells were significantly promoted. Besides that, after *KCNQ1OT1* knockdown, cell proliferation, colony formation, and cell invasion of OC cells were significantly inhibited. Collectively, our work indicated that *KCNQ1OT1* may have an oncogenic role in OC.

Bioinformatic analysis showed *MIR‐142‐5p* was a possible target for *KCNQ1OT1*. *MIR‐142‐5p* was reported to be a decreased expression in non‐small cell lung cancer tissues and cells (Wang, Liu, Fang, & Yang, 2017). Overexpression of *MIR‐142‐5p* suppressed non‐small cell lung cancer progression in vitro and in vivo via regulating phosphatidylinositol‐4,5‐bisphosphate 3‐kinase, catalytic subunit alpha (171,834) (Wang et al., 2017). Moreover, *MIR‐142‐5p* was revealed to function as a bridge between *KCNQ1OT1* and cyclin‐dependent kinase 5 (123,831) to regulate lung adenocarcinoma cell migration, invasion, and epithelial mesenchymal transition (Jia et al., 2019). In this work, we showed *MIR‐142‐5p* expression was decreased in OC cells and correlated with poor overall survival of OC patients. Luciferase activity reporter assay revealed the direct interaction of *MIR‐142‐5p* and *KCNQ1OT1*. Moreover, by exploring the target of *MIR‐142‐5p*, we identified a direct connection of *MIR‐142‐5p* and *CAPN10*. *CAPN10* was found highly expressed in OC cells and predicted poor overall survival of cancer patients. *CAPN10* belongs to the mitochondrial calpain system and reported to be regulated by oncogene *GAEC1* (612,130) to promote tumor progression (Chan et al., 2013). One drawback of this work was the lack of in vivo experiments. Hence, we will perform animal experiments in the near future to validate the *KCNQ1OT1*/*MIR‐142‐5p*/*CAPN10* axis in OC and to confirm their roles in regulating cancer progression.

To sum up, our results revealed that *MIR‐142‐5p*, sponged by *KCNQ1OT1*, target *CAPN10* to affect the progression of OC. Our study provided novel insights on the understanding of OC progression and the potential to use *KCNQ1OT1* as a target for OC therapy. However, further studies are needed to understand the clinical significance of *KCNQ1OT1*/*MIR‐142‐5p*/*CAPN10* in OC.

## CONFLICT OF INTEREST

The authors declare that they have no conflict of interest.
